# Sleep as a driver of pre- and postnatal brain development

**DOI:** 10.1038/s41390-024-03371-5

**Published:** 2024-07-03

**Authors:** Eline R. de Groot, Jeroen Dudink, Topun Austin

**Affiliations:** 1 Department of Neonatology, Wilhelmina Children’s Hospital, University Medical Centre Utrecht, Utrecht, The Netherlands; 2 Brain Centre Rudolf Magnus, University Medical Centre Utrecht, Utrecht, The Netherlands; 3 NeoLab, Evelyn Perinatal Imaging Centre, The Rosie Hospital, Cambridge University Hospitals, Cambridge, UK

## Abstract

**Abstract:**

In 1966, Howard Roffwarg proposed the ontogenic sleep hypothesis, relating neural plasticity and development to rapid eye movement (REM) sleep, a hypothesis that current fetal and neonatal sleep research is still exploring. Recently, technological advances have enabled researchers to automatically quantify neonatal sleep architecture, which has caused a resurgence of research in this field as attempts are made to further elucidate the important role of sleep in pre- and postnatal brain development. This article will review our current understanding of the role of sleep as a driver of brain development and identify possible areas for future research.

**Impact:**

- The evidence to date suggests that Roffwarg’s ontogenesis hypothesis of sleep and brain development is correct.
- A better understanding of the relationship between sleep and the development of functional connectivity is needed.
- Reliable, non-invasive tools to assess sleep in the NICU and at home need to be tested in a real-world environment and the best way to promote healthy sleep needs to be understood before clinical trials promoting and optimizing sleep quality in neonates could be undertaken.

## Introduction

Humans sleep about one third of their lives and for infants this is even longer. By the time an infant turns one year old, it has spent more than half of its life asleep. The increased prevalence of sleep in infants, and especially neonates, suggests that sleep plays an important role in early development.

Recently, technological advances have enabled researchers to automatically quantify neonatal sleep architecture, which has caused a resurgence of research in this field, attempting to explore the potential role of sleep in pre- and postnatal brain development. This article will review the history of newborn sleep research, our current understanding of the important role of sleep as a driver of brain development and identify possible areas for future research.

## A brief history of infant sleep research

In January 2024, Kristina Denisova published an English translation of the 1926 article “Periodic phenomena in the sleep of children”^1^. This research, by Maria Denisova and Nicholai Figurin, is one of the first to report cyclical periods of increased respiration and eye and body motility during sleep in infants, a precursor to what is now known as rapid eye movement (REM) sleep and non-REM sleep (in newborn infants this is referred to as active and quiet sleep respectively). They also found that exogenous stimuli had different effects depending on the depth of sleep. In 1937, Isabelle Wagner published a similar study in which she described seven sleep stages, based on her observations of the reactivity of 197 infants to a total of 5342 stimuli^2^. Her classification also explicitly mentions movements, including ‘eyelid movements’.

These two studies precede the work from Kleitman & Aserinsky in the 1950s in which they studied adult eye movements^3^ and infant motility cycles^4^ during sleep. In the latter study, Aserinsky distinguished two major stages: (1) “No Eye Movement periods”, which lasted 20–25 min for all infants and (2) an “active portion of the motility cycle”. The length of this “active period” showed high inter- and intraindividual variability. Aserinsky mentioned that the underlying mechanism behind this motility cycle is unclear but might be due to “an inherent rhythm within the CNS”.

Another famous description of infant sleep cycles and their underlying mechanisms came from Peter Wolff in 1959^5^. Wolff described regular and irregular sleep, based primarily on respiration rate. During sleep, Wolff observed a variety of spontaneous startles and other movements, the quality and quantity of which differed between sleep stages. Wolff hypothesized that these spontaneous movements during sleep were caused by “spontaneous activity of the central nervous system”^5^. Howard Roffwarg^6^ repeated and further substantiated this hypothesis in 1966, informed by emerging knowledge about the role of sleep and more specifically of the two sleep stages (REM and non-REM sleep) that were distinguished by Kleitman and Aserinsky^3^.

Roffwarg describes the high ratio of REM sleep in newborns, which diminishes rapidly over time^6^. According to Roffwarg “The early large percentages of REM sleep compel us to look to early development for the most important function of REM sleep”. At this time it was already known that during REM sleep “the pontine area sends impulses to motor as well as to sensory areas of the brain. After reaching the thalamus from the pons, the impulses appear to traverse the usual pathways to cortex”^6^. Based on this knowledge Roffwarg molded the ontogenic sleep hypothesis, relating neural plasticity and development to REM sleep, a hypothesis that current fetal and neonatal sleep research is still exploring:

“(REM sleep in newborns) might assist in structural maturation and differentiation of key sensory and motor areas within the central nervous system, partially preparing them to handle the enormous rush of stimulation provided by the postnatal milieu, as well as contributing to their further growth after birth.”^6^

Following these early observations and hypotheses, a number of standardized methods to assess infant sleep and behavior have emerged (for an extensive review on this topic, see Bik et al^7^.). Furthermore, recent developments in the field of machine learning have enabled continuous sleep stage assessment using physiological parameters to be made unobtrusively at the cotside^8–10^.

### Box 1 Early sleep architecture

During the first few days of its life, a healthy term newborn infant spends most of the time asleep, of which on average just over 50% is spent in active sleep (AS; i.e. the neonatal equivalent of REM sleep)^11^. However, total sleep time rapidly decreases, reaching an average of 12–15 h per day by one month^12,13^. At this time, AS still makes up the majority of the sleep cycle (50–80%)^13^. Over the course of the first year, sleep architecture changes, with AS-onset giving way to quiet sleep (QS; i.e. the neonatal equivalent of non-REM sleep) onset and the percentage of AS decreasing to less than 50%^13^.

Prenatally, behaviors which resemble sleep cycles emerge in fetal life from mid gestation and become more apparent from 32 weeks’ gestational age (GA)^14^. Similarly, in preterm infants, basic sleep cycling can be seen from 24–30 weeks’ GA, which again is better defined after 32 weeks GA^15,16^. Preterm infants spend around 16–22 h per day asleep, with 40–60% spent in AS^16–19^. The wide range of reported AS in the preterm infant reflects the amount of sleep investigators classified as indeterminate sleep,

Besides general sleep architecture, changes can be seen in the type of cortical activity measured from an electroencephalogram (EEG). The preterm EEG is visibly immature and defined by alternations between continuous and discontinuous patterns, which are associated with AS and QS respectively^15^. EEG patterns become more distinctly related with sleep stages as preterm infants mature.

From 43-48 weeks post menstrual age (PMA) sporadic spindle-like activity is visible during QS^15^ and around 3 months term equivalent age (TEA) definitive sleep spindles appear^20^. Over time, patterns of AS and QS slowly mature and are replaced by REM sleep and non-REM sleep respectively, at around 3–5 months of age which is reflected in the cortical EEG^21^, with sleep spindles occurring during non-REM/QS^20^. ^20^By 5–8 months, EEGs of non-REM stages show clear signs of slow waves, with delta bands of 0.5–4.0 Hz and sleep spindles of 7–14 Hz^21^.

## Emerging confirmation of Roffwarg’s ontogenesis hypothesis

In both preterm humans and newborn animals, AS is characterized by patterned, endogenously generated, spontaneous activity^22,23^. In rodents such spontaneous activity has been shown to be essential for cortical organization and development of thalamocortical connectivity^24^.

In humans, similar activity can be visualized using EEG and has been described as ‘spontaneous activity transients’ (SATs)^22^. SATs first appear in the preterm EEG by 24 weeks PMA and are most easily recognized as a sudden burst of high amplitude activity^22^. SATs are considered a hallmark of the premature EEG and are most frequently observed during AS until 33 weeks PMA^25^. SATs are thought to be involved in the establishment and survival of both thalamocortical sensory pathways and cortico-cortical connections^26^.

**Fig. 1:**
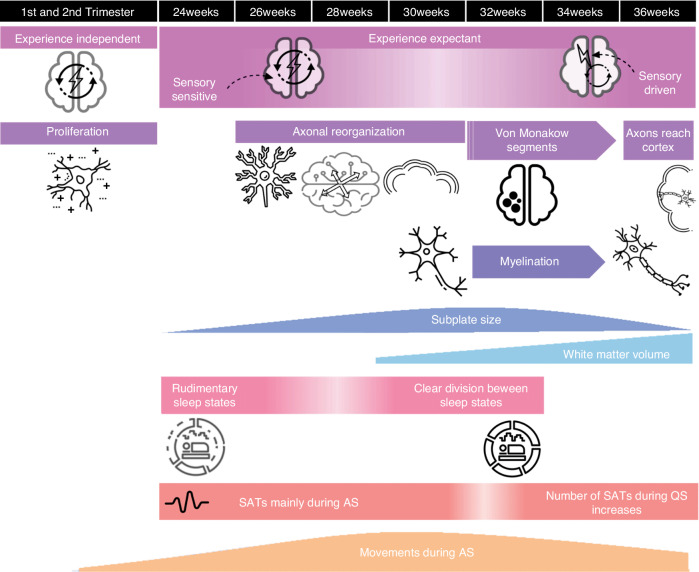
Sleep architecture and early brain development. An overview of neurodevelopmental processes and changes in sleep architecture during early development.

SATs can be triggered by endogenous mechanisms in the subplate—a transient layer in the developing brain that serves as the ‘waiting room’ for developing neurons—by spontaneous sensory input (i.e. through endogenously generated motor activity) or by extrinsic sensory input^26,27^. Spontaneous sensory input is produced from 10–12 weeks PMA onward and upregulated from 15-16 weeks PMA^28^. Behaviorally, this endogenous activity manifests as twitches. In the last trimester, the number of movements seems to either decline (30–36 weeks PMA)^29,30^ or remain stable (32–36 weeks PMA)^31^. Movement amplitude increases in this period (30–36 weeks PMA)^29,31^. An overview of the parallel development of movements and SATs during sleep is shown in Fig. 1.

### Experimental studies

It has been suggested that twitches in utero provide the sensory stimulation necessary to develop cortical body maps in the somatosensory cortex^27^. This mechanism has been further explored in rodent studies, mainly by the group of Mark Blumberg (e.g. refs. ^32,33^). It is important to note that rodent brain development before birth is slightly delayed compared to humans. The rat brain reaches the level of maturation of a term newborn at 10 days after birth^34–36^. Until the 8^th^ postnatal day, SATs in rat pups manifest both localized and large-scale waves. As opposed to the spatially restricted activity that occurs during wakefulness, these large-scale waves are only seen during early life sleep. This distinction suggests that sleep plays a critical role in the development of activity-dependent neural circuits^37^. Moreover, in rat pups, twitches are mainly present during the first two weeks of postnatal life^38,39^. Finally, in this period SATs are more easily triggered by AS-related twitches compared to wake-related movements^40^.

To further elucidate the association between SATs and twitches, Blumberg et al. followed the neural pathway of the neonatal rat twitch^33^. The twitches are generated in the red nucleus and project directly onto motor neurons in the spinal cord controlling the fore- and hindlimbs. From the spinal cord, reafferent sensory feedback flows to the external cuneate nucleus and from there to the cerebellum, thalamus and primary somatosensory (S1) and motor (M1) cortex, finally ending in the hippocampus. These findings support the hypothesis that these endogenous signals are specifically attuned to support the development of the somatosensory and motor homunculus. Similar findings in the visual system have shown that spontaneous retinal waves trigger bursts of neural activity in downstream structures, including the visual cortex, primarily during sleep^41^.

The importance of sleep for neurodevelopment is highlighted by findings that show how active wakefulness interferes with the sensory stimulation that is essential for development^42^. For example, in 9- and 12-day old rat pups, cortical activity that was associated with retinal waves was suppressed by both spontaneous and evoked awakenings^41^. In other words, pre- and early postnatal neurodevelopment is mainly supported by cortical activity that occurs during sleep.

### Human studies

Research in human infants both confirm and expand on the findings in experimental studies. For example, quality and quantity of endogenously generated movements in human fetuses, preterm infants and term neonates have been associated with behavioral and neurological outcome as children develop^43^. Furthermore, increased numbers of SATs have been linked to larger brain volumes in human preterm infants^44–46^.

There is a growing body of work relating sleep states to distinct functional connectivity network dynamics. Using EEG, Tokariev et al, have shown that there is a reorganization of functional brain networks during the transition from QS to AS and this reorganization is attenuated in preterm infants and predicts visual performance at 2 years^47^. Uchitel et al, using high density diffuse optical tomography (HD-DOT) found stronger interhemispheric connectivity during AS relative to QS, with stronger short-range connections in QS relative to AS in healthy term infants^48^.

Furthermore, more AS between 29- and 32-weeks PMA was associated with increased total brain volume and white matter volume, while ventricular volume was decreased^49^. More specifically, the brain volumes of the left frontal and occipital lobes were increased. Brain volume^46,50^ and connectivity^51^ of the frontal regions is often impaired due to preterm birth. The association between more AS and increased volume in these regions implies a positive influence of AS on the development of neural regions that are most affected.

## Changes in sleep architecture over the course of early neurodevelopment

While sleep is important throughout development, the role of sleep changes during different stages of development, adapting to the developmental needs of the body. Consequently, sleep architecture and the characteristics of sleep parameters change, including for example the quantity and pattern of twitching and retinal activity in animals^52^ and fetuses^53^.

When looking at the developmental trajectory of spontaneous neural activity, it stands out that before 33–34 weeks PMA in human infants (and before 8 postnatal days in rats; P8), SATs occur mainly during AS. After this period, the number of SATs during QS increases, as well as the relative amount of QS^15,19,25^. Although the exact role of QS is not fully understood in this developmental stage, the adult equivalent of QS (non-REM sleep) is involved in, among other things, synaptic downscaling in order to facilitate efficient network formation^54^.

The emerging importance of QS—highlighted by the shift to more SATs during this stage seems to indicate a shift in the role of sleep in general. Experimental research supports this notion. For example, in rats before P10, the primary motor cortex (M1) only shows activation as a result of endogenous stimulation during sleep and not during wake movements^33,42^. However, after P12, 80% of M1 neurons respond specifically to movements during wakefulness and responses to twitches during sleep are inhibited^33^. When linking these changes in sleep parameters to neurodevelopment, one specific event stands out. A major shift in neurochemistry that occurs around P10 in rats and the last trimester in humans, namely the ‘GABA-shift’.

### The GABA-shift

In adult humans and rats, gamma-aminobutyric acid (GABA) is the main inhibitory neurotransmitter in the central nervous system; GABA_A_ receptors are ligand-activated chloride channels, with an inward flux of chloride causing hyperpolarization and therefore increasing the threshold of activity needed to excite a neuron. However, in early development activation of GABA receptors leads to an efflux of chloride from neurons which has a depolarizing effect—i.e. facilitating excitation of neurons. During this period depolarizing GABA plays an essential role in the coordination and timing of key prenatal neurodevelopmental processes, including spontaneous activity^22,55^, arborization, synapse formation and (pre-)myelinization.

After the ‘GABA-shift’, when GABA switches to having a hyperpolarizing effect, the inhibitory quality of the neurotransmitter can facilitate the attunement of the postnatal brain to the specific requirements of the outside environment^56^. In other words, the brain seems to be more attuned to endogenous stimulation when GABA has a depolarizing effect, and it is more attuned to exogenous stimulation when GABA has a hyperpolarizing effect. In the context of behavioral states, sleep and endogenously generated activity during sleep might be more beneficial to the developing brain before the GABA-shift, whereas waking activity and exogenous sensory stimulation may be more beneficial after the GABA-shift. Nevertheless, in rat pups, myoclonic twitches and endogenous brain activity still occur during AS after the ‘GABA-shift^32,42^. Thus, it is possible that both exogenous stimulation and endogenously generated brain activity have a developmental role after GABA becomes hyperpolarizing.

If the brain has not undergone the GABA-shift yet, which may be the case in preterm infants, an increase of exogenous sensory stimulation—such as during waking activity – might disrupt the ongoing neurodevelopmental processes and consequently the development of brain structures. However, if the brain has already gone through the GABA-shift, a combination of increased sensory stimulation and sufficient sleep might best facilitate neurodevelopment. Knowledge about the exact timing of the GABA-shift is therefore essential to be able to provide an appropriate neuroprotective environment for developing preterm infants.

### Timing of the GABA-shift

Unfortunately, the exact timing of GABA shifting from depolarizing to hyperpolarizing in humans remains unclear. Based on both experimental and human research, the shift seems to happen in the third trimester^23,56–59^ or just after birth^22,23,56^, although there appears to be regional heterogeneity in GABA receptor maturation, with, for example the hippocampus maturing earlier than other brain regions^60^. Besides cell type, sex and brain region, Peerboom and Wierenga^56^ have identified several factors that may influence the timing of the GABA-shift. The general idea comprises an intrinsic developmental program that might be affected by molecular and environmental factors. Although it is currently unclear to what extent preterm birth influences the GABA-shift^61,62^, preterm birth might decrease exposure to molecular factors that are thought to repress the GABA-shift, while increasing the exposure to environmental factors that are thought to induce the GABA-shift. Indeed, recent studies suggest a heightened responsivity of the preterm brain to exogenous stimuli^63–65^ —implying that the timing of the GABA-shift might be altered in very preterm infants.

However, neurophysiological studies assessing the developmental trajectory of twitches and associated neural activity in preterm infants, challenge the hypothesis of an early GABA-shift. Between 31- and 42-weeks GA, isolated hand movements during AS induce alpha-beta oscillations with a specific somatotopic distribution^66^. As term equivalent age approaches, the alpha-beta oscillations decline and fully disappear by 41 weeks GA, while increase in the delta oscillations remains. This pattern resembles the pattern found in rodents^33,67^ and might serve as an indication that preterm birth might not result in a change in the timing of the GABA-shift.

To optimally support the sleep cycle and provide appropriate stimulation for the developing preterm brain, it is necessary to further understand the timing of the GABA-shift in preterm and term-born infants.

## The association between sleep and neonatal illness

A growing body of evidence confirms Roffwarg’s ontogenesis hypothesis that sleep is an essential driver of early brain development. Furthermore, the changes in sleep architecture and characteristics may provide insight into the neurodevelopmental trajectory of high-risk infants.

Both sleep and neurodevelopment are affected in the case of early pathology, whether this involves preterm birth, neonatal encephalopathy, congenital disorders or any other illnesses in the neonatal period. It is believed that the alteration in sleep structure following preterm birth is caused by a combination of factors, including exposure of the inherently immature nervous system to the external environment and comorbidities associated with preterm birth, such as discomfort or pain^68^. In the term infants neural insults—such as hypoxic ischemic encephalopathy—influence sleep-wake cycling in the neonatal EEG^69^. Furthermore, less QS and more AS was observed in asphyxiated infants^70^. Finally, brain injury has been associated with a later onset of QS after birth, which is considered a marker of the beginning of sleep cycling^71^.

As development proceeds, cerebral palsy and other forms of acquired brain injury are associated with sleep disturbances^72^ and increased asymmetry in sleep spindle spectral power between hemispheres^73^. These asymmetries may be directly related to structural damage sustained, which in turn could result in sleep disturbances.

Other neurodiverse conditions, such as autism spectrum disorder, are also known to be associated with sleep disturbances^74^. Whether sleep disturbances result from the underlying condition or exacerbate the condition remain unclear^75^. Other conditions that are associated with preterm birth, such as bronchopulmonary dysplasia (BPD), are associated with decreased sleep quality due to obstructive sleep apnea at a later age^76,77^. Besides this direct association with BPD, a complex interplay between respiratory pathology and neurodevelopmental problems in children born extremely preterm may result in sleep-disordered breathing symptoms and sleep problems in childhood^78^.

In summary, sleep patterns and neurodevelopment are clearly entwined in early development. Preterm sleep patterns may therefore both serve as an indicator of current neurodevelopmental status and present an opportunity for interventions aimed to support preterm neurodevelopment^68^.

## Current dilemmas and future research

The final piece of Roffwarg’s puzzle is the question whether impaired sleep causes impaired neurodevelopment or if sleep is just a marker of the current neurodevelopmental state of an infant. To assess if improving sleep quality is a valid and effective neuroprotective strategy, ideally a randomized controlled trial should be conducted, although this would be challenging to carry out.

In order to improve sleep quality in the NICU and at home, several barriers need to be overcome. First, the research community should consider the best way to monitor sleep. Due to technological advances, it has now become feasible to continuously monitor preterm sleep stages unobtrusively^9,79–82^. However, these methods use a variety of modalities with variable quality in terms of reliability and validity in detecting sleep states.

Sleep is defined and classified by a constellation of behavioral, physiological and neurophysiological phenomena, rather than direct measurements of the key processes occurring in the brainstem and thalamus. Most preterm sleep classifications are based upon behavioral assessments alongside EEG and cardiorespiratory monitoring. However, most current algorithms that continuously monitor sleep stages use only one modality, reducing both their validity and reliability. Limiting dimensionality in sleep assessment has the potential of introducing confounders. To circumvent such problems, it may be preferable to develop algorithms that are used for research purposes based on multiple modalities.

Once a consensus has been achieved on monitoring sleep, there comes the question of how to define ‘good quality sleep’. There is a complex interplay between the neurobiological needs of the developing brain and the external environment. A better understanding of the relationship between sleep and functional brain development is needed. While neural activity during AS seems to be the most important driver of early brain development, only protecting AS at the expense of QS may not necessarily be the best course of action. Moreover, the literature about the ‘GABA-shift’ suggests that sensory stimulation may aid further development depending on the developmental stage.

Finally the impact of different environmental phenomena on sleep needs to be better understood. A great deal of research has been done investigating the acoustic environment within the NICU. However, it should be noted that the fetus does not develop in a silent environment, and nor should the preterm neonate. However, a better understanding is needed of how different acoustic stimuli—both pleasant (e.g. music therapy) or noxious (e.g. alarms) impact sleep and the sleep-wake cycle.

Developing a more personalized approach to ‘good sleep hygiene’, tailored to the infant’s stage of development, underlying pathologies and family situation both in the NICU and at home is likely to reap the most rewards. However, developing a robust evidence base is one of the biggest challenges currently in this field.

## Conclusion

The evidence to date suggests that Roffwarg’s ontogenesis hypothesis of sleep and brain development is correct and that alterations in sleep and sleep-wake cycling are associated with a range of neurodevelopmental and neurodiverse conditions, including prematurity. However, many questions remain unanswered. A better understanding of the relationship between sleep and the development of functional connectivity is needed. Reliable, non-invasive tools to assess sleep in the NICU and at home need to be tested in a real-world environment and the best way to promote healthy sleep needs to be understood before clinical trials promoting and optimizing sleep quality in neonates could be undertaken. Finally, promoting healthy sleep beyond the neonatal period, for both the infants and their caregivers is important to maximize beneficial outcomes.
